# Identifying Medication-Related Intents From a Bidirectional Text Messaging Platform for Hypertension Management Using an Unsupervised Learning Approach: Retrospective Observational Pilot Study

**DOI:** 10.2196/36151

**Published:** 2022-06-29

**Authors:** Anahita Davoudi, Natalie S Lee, ThaiBinh Luong, Timothy Delaney, Elizabeth Asch, Krisda Chaiyachati, Danielle Mowery

**Affiliations:** 1 Department of Biostatistics, Epidemiology, and Informatics Institute for Biomedical Informatics University of Pennsylvania Philadelphia, PA United States; 2 Division of General Internal Medicine Department of Medicine The Ohio State University Wexner Medical Center Columbus, OH United States; 3 Penn Medicine Predictive Healthcare University of Pennsylvania Health System Philadelphia, PA United States; 4 Center for Health Care Innovation University of Pennsylvania Philadelphia, PA United States; 5 Leonard Davis Institute of Health Economics University of Pennsylvania Philadelphia, PA United States; 6 Department of Medicine Perelman School of Medicine University of Pennsylvania Philadelphia, PA United States

**Keywords:** chatbots, secure messaging systems, unsupervised learning, latent Dirichlet allocation, natural language processing

## Abstract

**Background:**

Free-text communication between patients and providers plays an increasing role in chronic disease management, through platforms varying from traditional health care portals to novel mobile messaging apps. These text data are rich resources for clinical purposes, but their sheer volume render them difficult to manage. Even automated approaches, such as natural language processing, require labor-intensive manual classification for developing training data sets. Automated approaches to organizing free-text data are necessary to facilitate use of free-text communication for clinical care.

**Objective:**

The aim of this study was to apply unsupervised learning approaches to (1) understand the types of topics discussed and (2) learn medication-related intents from messages sent between patients and providers through a bidirectional text messaging system for managing participant blood pressure (BP).

**Methods:**

This study was a secondary analysis of deidentified messages from a remote, mobile, text-based employee hypertension management program at an academic institution. We trained a latent Dirichlet allocation (LDA) model for each message type (ie, inbound patient messages and outbound provider messages) and identified the distribution of major topics and significant topics (probability >.20) across message types. Next, we annotated all medication-related messages with a single medication intent. Then, we trained a second medication-specific LDA (medLDA) model to assess how well the unsupervised method could identify more fine-grained medication intents. We encoded each medication message with n-grams (n=1-3 words) using spaCy, clinical named entities using Stanza, and medication categories using MedEx; we then applied chi-square feature selection to learn the most informative features associated with each medication intent.

**Results:**

In total, 253 participants and 5 providers engaged in the program, generating 12,131 total messages: 46.90% (n=5689) patient messages and 53.10% (n=6442) provider messages. Most patient messages corresponded to BP reporting, BP encouragement, and appointment scheduling; most provider messages corresponded to BP reporting, medication adherence, and confirmatory statements. Most patient and provider messages contained 1 topic and few contained more than 3 topics identified using LDA. In total, 534 medication messages were annotated with a single medication intent. Of these, 282 (52.8%) were patient medication messages: most referred to the medication request intent (n=134, 47.5%). Most of the 252 (47.2%) provider medication messages referred to the medication question intent (n=173, 68.7%). Although the medLDA model could identify a majority intent within each topic, it could not distinguish medication intents with low prevalence within patient or provider messages. Richer feature engineering identified informative lexical-semantic patterns associated with each medication intent class.

**Conclusions:**

LDA can be an effective method for generating subgroups of messages with similar term usage and facilitating the review of topics to inform annotations. However, few training cases and shared vocabulary between intents precludes the use of LDA for fully automated, deep, medication intent classification.

**International Registered Report Identifier (IRRID):**

RR2-10.1101/2021.12.23.21268061

## Introduction

### Background

Digital health technology has enabled patient-clinician communication to move beyond the confines of a face-to-face clinician-patient encounter. For years, the patient portal has been the primary route of electronic patient communication with providers [1-5]. Increasingly, there are mobile health apps that also enable communication between patients and providers through SMS, particularly for management of chronic conditions like hypertension [6,7]. SMS platforms add to the increasing number of channels for facilitating low-threshold, frequent, asynchronous communication between patients and clinical teams. However, enhancing communication pathways also adds burden to clinical teams, which are often already struggling to manage patient message volume through more conventional paths, such as the patient health portal. These pathways can be a source of clinician burnout due to technostress, time pressure, and workflow-related issues [8,9].

Despite the importance of patient message data for clinical care, the current state for clinical review of patient message data is largely manual [10,11]. Natural language processing (NLP) and machine learning (ML)–based systems could facilitate triaging of messages to the right clinical sources (providers, nurses, medical assistants, billing, etc) for appropriate decision-making [12], thereby minimizing the burden of messages on already-strained personnel.

### Natural Language Processing and Digital Health Technology

There are some promising examples in the literature of NLP and supervised ML approaches to more efficiently filter and review messages for clinical purposes [13-15]. Chen et al [15] developed HypoDetect (Hypoglycemia Detector) to automatically identify hypoglycemia incidents within messaging threads reported by US veterans with diabetes through SMS. They trained and tested three supervised ML algorithms to classify each thread as containing a hypoglycemia incident (positive) or not (negative). Liu et al [14] trained and tested a deep learning approach—Longformer-masked language models using Hugging Face—to classify conversation stages of messages and behaviors present in messages from both texters and volunteers. Stenner et al [13] developed PASTE (Patient-Centered Automated SMS Tagging Engine), a rule-based NLP system for encoding medication-related messages from MyMediHealth, which is a medication management system for scheduling and administering medications and sending reminders to patient cell phones. Although all three examples cited are promising ways to harness NLP and ML for clinical patient-facing applications, a large amount of labeled data is necessary to train and test robust NLP or ML models. Stenner et al [13] focused narrowly on NLP for medication-related concepts and, therefore, used existing libraries of annotated data. However, this strategy is not possible for identification of novel intents. Thus, in the cases of HypoDetect and Shout, which represented the application of ML to novel topics, researchers manually annotated 3000 and 8844 messages, respectively, to begin training their models.

Unsupervised ML approaches have been shown to subgroup texts with similar word usage and could reduce the burden of manual annotation for training clinical models for intent classification. Therefore, we investigated the utility of unsupervised methods for deriving message intents, in particular, latent Dirichlet allocation (LDA). LDA is an unsupervised, generative statistical model for learning subgroups of observations within a data set based on similarities among observations [16]. LDA has been leveraged to derive insights into patient communication data in patient portals [17,18], but its usage to classify message intent, particularly in mobile text messaging technologies, has been largely unexplored. The purpose of this proof-of-concept study was to explore the extent to which LDA might be applied to patient-provider messages to accurately identify domains (ie, topics) of clinical relevance (ie, those topics that were deemed informative for clinical action that are indicative of intents). In this study, we applied LDA to a database of patient-provider messages exchanged in a mobile app designed for remote hypertension monitoring and examined the usability of LDA-derived topic classes for identifying and classifying novel intents.

## Methods

### Overview

**Figure 1:**
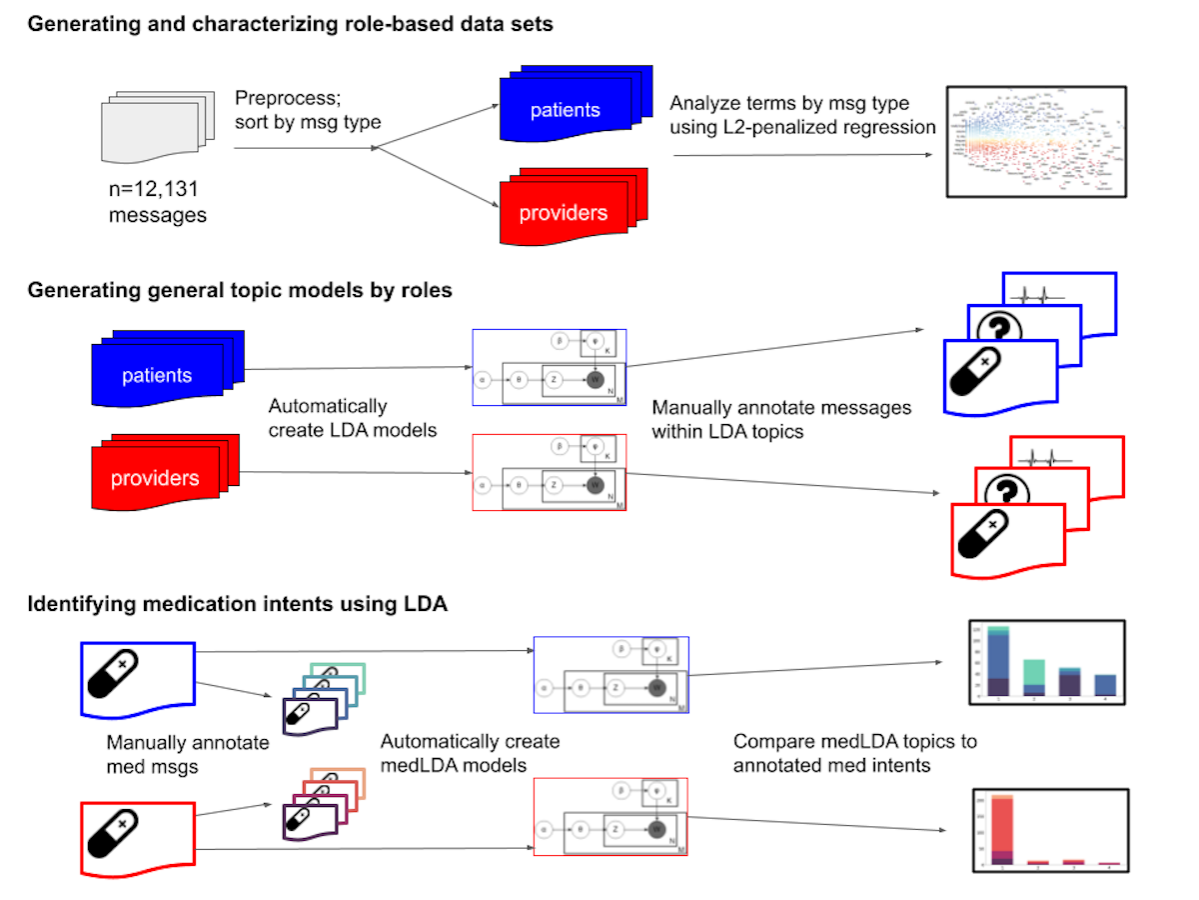
Study workflow. LDA: latent Dirichlet allocation; med: medication; medLDA: medication-specific LDA model; msg: messages.

In this study, we retrospectively studied messages exchanged through a third-party mobile app. Participants were adults enrolled in Penn Medicine’s Employee Hypertension Management Program (eHTN) from 2015 to 2019. To protect the privacy of our study participants, all data were deidentified using a text deidentification system called De-Id prior to the study and the analyses [19]. In Figure 1, we outline our methodology, and we describe our analytical framework in subsequent sections.

### Ethics Approval

This study was approved by the Institutional Review Board at the University of Pennsylvania (approval No. 834667).

### Employee Hypertension Management Program

The eHTN is a primarily remote hypertension management program for Penn Medicine employees with uncontrolled hypertension. Through the eHTN, employees diagnosed with uncontrolled hypertension at an initial office visit receive a prescription medication, a treatment plan for blood pressure (BP) management, and a BP cuff for home-based measurements. A critical component of the program is out-of-office communication of BP readings, typically once every 2 weeks, plus unlimited bidirectional text message conversation between the clinical team and patients for issues related to BP management. These patient-clinician interactions were facilitated through a proprietary Health Insurance Portability and Accountability Act–compliant, bidirectional text messaging mobile app. Topics discussed reflected the spectrum of issues related to hypertension management, including questions about BP, medication-related questions or complaints, refill requests, and BP machine or cuff issues, as well as questions about the app itself. There were no technical restrictions to the conversation, though content that was less relevant to hypertension management (eg, a statin medication refill request) was generally redirected by the BP clinical team. Conversations could be initiated by patients (eg, a medication question) or by clinicians (eg, to request an updated BP reading). Our study period was the duration of app usage, from June 2015 to November 2019.

### Generating and Characterizing Role-Based Data Sets

To automatically identify topical themes by the roles of patients and providers, we classified messages into two data sets: inbound (ie, patient-generated messages) and outbound (ie, provider-generated messages). For each data set, we removed messages that appeared to be automatic messages generated by the app, such as patient enrollment (eg, “Registered to use the PROGRAM app”) or calendar events (eg, “Calendar event created: Plan Check-In”). Among inbound messages, patients could report BP readings as structured data elements (eg, “Annotation: Pulse 76”) and could add contextual information as free text (eg, “left arm”): for example, “Annotation: Pulse 95. Ran down the steps.” We removed all app-specific prefixes (eg, “Annotation:”) from the messages, but kept the remaining parts of the text messages in the model. Next, we preprocessed each message by changing words to lowercase (eg, “Meds” reduced to “meds”), removing stop words (eg, “of” and “the”), and stemming terms (eg, “scheduling” stems to “schedul”). To better understand commonality and differences between the patient and provider messages, we analyzed and visualized individual words between data sets according to their frequency and informativeness. We computed and visualized the word frequencies according to their scaled L2-penalized regression coefficients using scattertext [20].

### Generating LDA Models by Roles: Topics and Subtopics

Next, we aimed to automatically learn topics dispersed within each message data set. For each processed data set (ie, patient and provider independently), we applied LDA. LDA is an unsupervised, generative statistical model for learning subgroups of observations within a data set based on similarities among observations [16]. We leveraged LDA to identify subgroups of messages with similar term usage and derive topics that might correlate to high-value intents hypothesized a priori (eg, medication reorders and appointment scheduling requests). To generate useful LDA models, we experimented with hyperparameters of α and β by varying their values from .01 to 1, their symmetry parameters, and the number of topics from 5 to 75 [21]. Our goal was to optimize the topics such that they should be precise, but the words that compose a topic can be diverse and be comprised of most words in the corpus. After manual inspection, the parameters for each model were set as follows to provide the most precise and diverse range of semantically coherent topics: α was set as asymmetric to ensure that document-topic density would result in more specific topic distributions per document; β was set as symmetric to ensure that word-topic density would result in less specific word distributions per topic. We also limited the number of topics to 50 after observing that the exact composition of terms listed across models with topics over 50 were identical with near zero weights, suggesting that LDA was unable to identify any additional distinct topics beyond 50 topics. In LDA models, each unit of analysis (eg, each message) is assigned LDA-derived topics with associated probabilities. For each message, the probabilities of each topic sum to 1; therefore, a message could have one or more significant subtopics. For example, a message may be assigned LDA topics 1, 2, and 5 with high probabilities associated with each topic. We defined a *primary topic* as the topic with the highest probability; a *secondary subtopic* was defined as any topic with a probability equal to or greater than .20. We chose a threshold of .20 based on the observation that most messages had single-digit probabilities or ones that were close to 0, and when messages contained multiple themes they often coincided with probabilities greater than .20. Note that a main topic can have a probability of less than .20 because some messages may or may not have a significant topic (ie, above .20 probability). Additionally, a given message could be assigned to two or more topics if both topics exceeded a probability of .20 (eg, if a message has a probability of including topic 15 of >.20 and topic 37 of >.20, the message will appear in both topics 15 and 37). For each message, we identified both primary and secondary topics.

### Comparing LDA-Generated Topics With Manually Derived Intents

To explore the clinical validity of the LDA-derived topics, we manually annotated a subset of messages with their intents, which is the term used to describe the goal or main idea of the text. The codebook for manual annotation was loosely based on an annotation schema from prior work on clinician-patient text communication [22]; briefly, two research team members (TL and NL) developed a common annotation codebook for messages exchanged through a different text message–based platform for remote hypertension management. The codebook was refined by review of over 1200 text messages exchanged in the program between October 1, 2020, and January 31, 2021; the review was conducted by our team members (TL and NL) for a separate internal pilot study. The codebook was further refined based on iterative discussion and review of the study text data by three team members (TL, NL, and DM). We attempted to apply this schema to each LDA-derived topic, but determined that the patient messages still contained heterogeneity (ie, 1 topic does not correlate to a single intent) and variability of intents.

Given these factors, we attempted to apply LDA to a more limited data set. We focused on the short messages that had a single LDA intent, occurred frequently, and appeared clinically useful; we chose medication-related intents for this last category, given the clinical importance of identifying medication-related communication. Each of three reviewers (TL, DM, and NL) manually reviewed the data set and annotated only those messages with a single intent related to medication; any messages that were deemed to have more than one intent were excluded from review, with the exception of messages where the second intent was a pleasantry. All medication messages were reviewed among the team to resolve disagreements through consensus. For all messages annotated with a single medication intent, we generated another medication-specific LDA (medLDA) model and attempted to reclassify the resulting messages according to the set k topics: k=4 for both patient and provider messages, based on manual annotation. Across each k topic, we report the majority class intent for that topic number and apply the heuristic of classifying each message within a particular topic to the majority class intent. We report the recall, precision, and F1 score of applying this heuristic for classifying each medication intent class [13,23].

### Visualizing Sublanguage of Medication Intents

We aimed to automatically capture the sublanguage of medication intents by identifying the most significant language features for each medication intent. To identify lexical features, each text message was preprocessed using spaCy by removing stop words, reducing case, and encoding n-grams. We applied term frequency–inverse document frequency to extract the most informative lexical features. To identify semantic features, we encoded the named entities of problems, treatments, and tests using the i2b2 (Informatics for Integrating Biology and the Bedside) named entity recognition (NER) model from the Stanza package in Python (version 3; Python Software Foundation) [24]. To standardize medication-related details, we encoded RxNorm categories and semantic medication categories of drug name, strength, route, frequency, form, dose amount, intake time, duration, dispense amount, refill, and necessity using the MedEx package [25]. Examples of semantic features can be found in Table 1. The stop words were removed in the preprocessing step, and question marks were represented by the word “question.” Within each subclass, we computed the word frequencies (n=1-3 words) and identified the most informative words using chi-square feature selection. We selected the lexical and semantic features (ie, n-grams, Stanza NER, and MedEx) that were most significantly associated with each medication intent (*P*<.05). We report the sublanguage features associated with each medication intent.

**Table 1:** Examples of lexical and semantic features.

Package type and category			Example^a^
**Stanza**			
	Problem	“Musinex, proair inhaler and delsym for *cough*”	
	Test	“Took meds between 8:30 and 9:00 am after my *MRI*^b^”	
	Treatment	“*Meds*^c^ taken late”	
**MedEx^d^**			
	Drug product name (DPN)	“Sorry Lisinopril was stopped *HCTZ*^e^ 25 mg added / daily Metoprolol decreased to 50 mg / daily.”	
	Drug ingredient (DIN)	“Sorry *Lisinopril* was stopped HCTZ 25 mg added / daily *Metoprolol* decreased to 50 mg / daily.”	
	Drug brand name (DBN)	“Hi have only 11 tablets of *amlodipine* left. Are you able to issue me a new prescription?”	
	Drug dose form (DDF)	“Dr doubled my Lisinopril and removed the water *pill* See OV^f^”	
	Dose (DOSE)	“Sorry Lisinopril was stopped HCTZ *25 mg* added / daily Metoprolol decreased to *50 mg* / daily.”	
	Dose amount (DOSEAMT)	“Hi have only *11 tablets* of amlodipine left. Are you able to issue me a new prescription?”	
	Frequency (FREQ)	“Sorry Lisinopril was stopped HCTZ 25 mg added / *daily* Metoprolol decreased to 50 mg / *daily*.”	
	Route (RUT)	“Lossrtan is giving me SOB^g^ chest pressure on *inhalation* is this l? Happens about 20 min after I take it and lasts thru day.”	
	Duration (DRT)	“Lossrtan is giving me SOB chest pressure on inhalation is this l? Happens about *20 min* after I take it and lasts thru day.”	
	Dose unit	“Good Morning can you call me in a refill for my bp^h^ *pills* please?”	

^a^Keywords are italicized.

^b^MRI: magnetic resonance imaging.

^c^meds: medications.

^d^The MedEx semantic type for each term is included in parentheses.

^e^HCTZ: hydrochlorothiazide.

^f^OV: office visit.

^g^SOB: shortness of breath.

^h^bp: blood pressure.

## Results

In this retrospective observational study, we studied text messages exchanged through a third-party mobile app between 253 participants who were enrolled in the eHTN and their clinicians. Of the patients who participated, 96.0% (243/253) were actively engaged in the program, sending at least one inbound message. Of the total 12,131 messages collected, 46.90% (n=5689) were generated by patients and 53.10% (n=6442) were generated by providers (Table 2).

**Figure 2:**
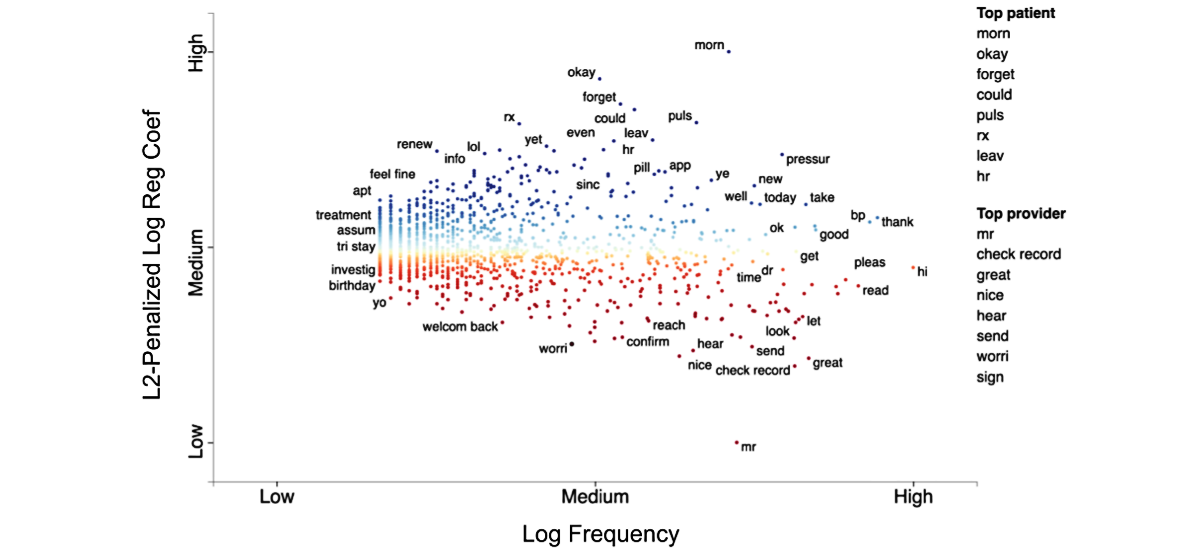
Characteristics of messages shown with a scatterplot image using word frequency and L2-penalized regression coefficients. Terms with higher usage are colored according to patients (blue) and providers (red). Terms with intermediate colors, such as green, yellow, and orange, reflect coefficients with values that have less of an association with patient or provider usage. Coef: coefficient; Reg: regression.

Using word frequency and L2-penalized regression coefficients, we identified distinct language use by role (ie, patient and provider; Figure 2). Within the patient messages (blue), we observed terms with higher coefficients and higher frequency, including temporal expressions (eg, “morning,” “evening,” “tonight,” “gm” [good morning], and “hr” [hour]), medical terms (eg, “rx” [prescription] and “pulse”), and confirmations (eg, “okay” and “thx” [thanks]). Within the provider messages (red), we observed terms with lower coefficients and higher frequency including salutations (eg, “mrs” and “mr”), directive verbs (eg, “check record,” “sign,” “send,” “confirm,” “recheck,” and “look”), and positive and negative sentiment (eg, “nice,” “great,” and “worry”).

**Figure 3:**
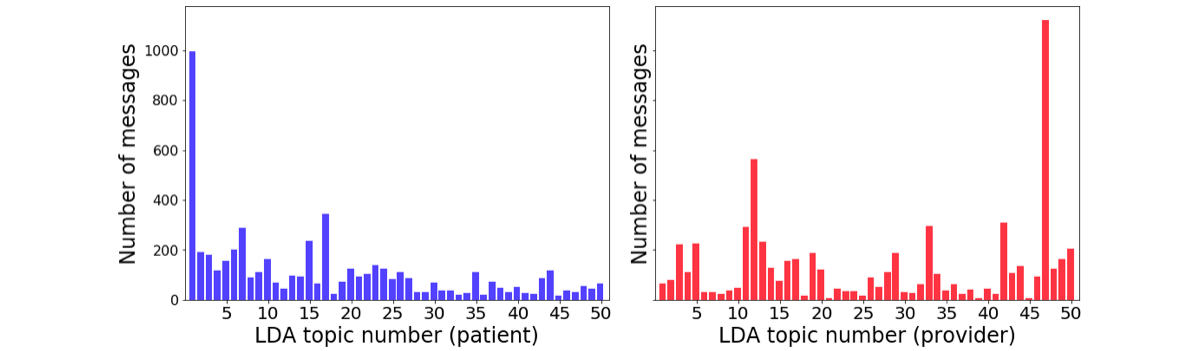
Distribution of patient (left) and provider (right) messages according to major topics. LDA: latent Dirichlet allocation.

When we applied LDA to the patient and provider text data sets, we observed a broad distribution of messages, with certain topics occurring more frequently for both patients and providers (Figure 3 and Table 3). In Figure 3, among the 5689 patient messages, the majority of messages occurred within topic 1 (n=1117, 19.63%; eg, “thank,” “ok,” “great,” and “nice”), topic 17 (n=412, 7.24%; “pulse,” “mg,” “daili” [daily], “take,” “tab” [tablet], “dose,” “losartan,” and “amlopidin” [amlodipine]), and topic 7 (n=395, 6.94%; “bp,” “read,” “hi,” “record,” “check,” and “today”). Among the 6442 provider messages, the majority of messages occurred within topic 47 (n=1249, 19.39%; “record,” “please,” “bp,” “update,” “read,” “hi,” and “check”), topic 12 (n=665, 10.32%; “good,” “look,” “work,” “bp,” “keep,” and “great”), and topic 42 (n=419, 6.50%; “call,” “schedul” [schedule], “hi,” “appt” [appointment], “please,” “appoint” [appointment], “come,” and “see follow” [see at follow-up]).

In Table 3, among 5689 patient messages, the majority were assigned 1 significant topic (n=2851, 50.11%), but it was also common for messages to be more heterogeneous, with 2 (n=1893, 33.27%) or 3 (n=564, 9.91%) co-occurring primary and secondary topics (Figure S1 in Multimedia Appendix 1). Similar heterogeneity was observed among provider messages; the majority of the 6442 messages were assigned to 1 topic (n=3311, 51.40%), but many had 2 (n=2466, 38.28%) or 3 (n=503, 7.81%) co-occurring topics (Figure S2 in Multimedia Appendix 1).

**Table 2:** Statistics according to patient and provider messages.

Message type	Patient messages (n=5689), mean (SD)	Provider messages (n=6442), mean (SD)
Words per message	17.01 (23.40)	26.75 (28.79)
Sentences per message	2.71 (2.02)	3.11 (2.11)
Messages per user	23.84 (26.22)	521.83 (1588.20)

**Table 3:** Distribution of patient and provider messages according to shared significant subtopics within each main topic.

Number of LDA^a^ topics by data set			Messages, n (%)
**Patient (n=5689)**			
	1	2851 (50.11)	
	2	1893 (33.27)	
	3	564 (9.91)	
	4	49 (0.86)	
	5	0 (0)	
**Provider (n=6442)**			
	1	3311 (51.40)	
	2	2466 (38.28)	
	3	503 (7.81)	
	4	22 (0.34)	
	5	0 (0)	

^a^LDA: latent Dirichlet allocation.

In Table 4, we depict the distribution of messages that were manually classified according to medication intent. Among the 282 patient medication messages, the intent of the majority of messages was medication request (n=134, 47.5%), followed by medication taking (n=79, 28.0%) and medication location (n=54, 19.1%). Among the 252 provider medication messages, the intent of the majority of messages was medication question (n=173, 68.7%), followed by medication question response (n=41, 16.3%). We observed lexical and semantic sublanguage features associated with each medication intent category according to the patient and provider data sets. Among the patient medication intents, for the medication location intent, terms associated with drug dispensaries (eg, “pharmacy” and “apothecary”), hospitals (eg, “hup” [Hospital of the University of Pennsylvania]), and street locations (eg, “Spruce” and “Market”) were common. For the medication question intent*,* we observed semantic types associated with MedEx drug names (eg, “DPN” [drug product name], “DBN” [drug brand name], “DDF” [drug dose form], and “DIN” [drug ingredient]), course (eg, “start” and “stop”), use of a question mark, and Stanza problem entity. The medication request and medication-taking intents commonly included MedEx categories (eg, “DOSE” and “FREQ” [frequency]), terms for refills and verbs (eg, “need” and “taking”), and Stanza treatment entities. Among the provider medication intents, for the medication change intent, terms associated with temporal expressions (eg, “tomorrow” and “week”), MedEx categories (eg, “DBN” and “DOSE”), and program references were common. Among the medication question and medication refill question intents*,* we observed terms associated with refill requests (eg, “refill,” “refilled,” and “need”), use of a question mark, and Stanza treatment entity. Among the medication question response intents, we observed terms associated with references and change (eg, “baseline” and “increasing”) as well as side effects.

**Figure 4:**
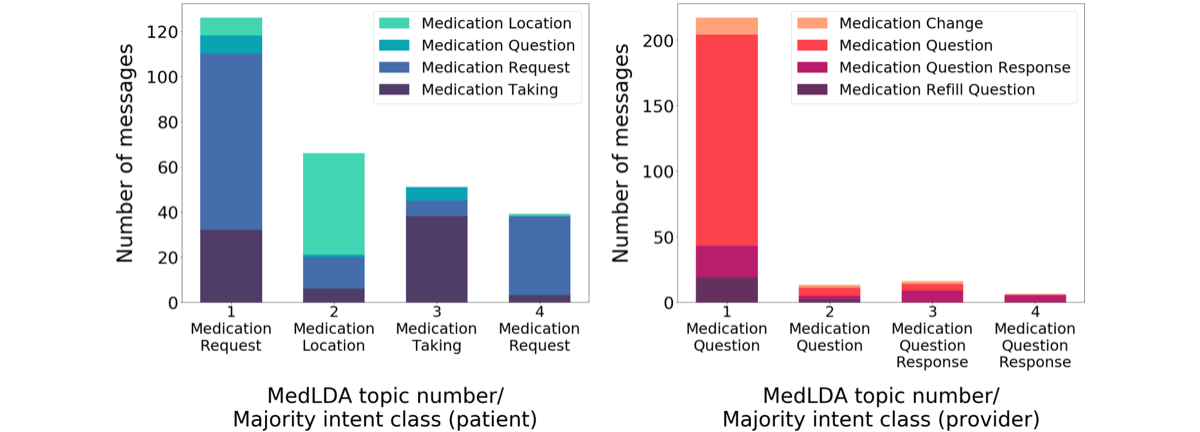
Distribution of medication intents among patient messages (left) and provider messages (right) in the medLDA model. medLDA: medication-specific latent Dirichlet allocation.

In Figure 4, we depict the outcomes of applying the medLDA model for both patient and provider messages. Among the patient messages manually classified according to medication intent and automatically classified within a topic, we observed a majority medication intent within each topic: medication request (topic 1), medication location (topic 2), medication taking (topic 3), and medication request (topic 4). Among the provider messages, the majority of medication intents within each topic were medication question (topics 1 and 2) and medication question response (topics 3 and 4).

In Table 5, by applying a majority intent class heuristic to classify each message within a topic number, we computed performance for predicting each medication intent category by message type. For patient messages, we observed high recall and moderate precision for medication location (recall=0.833; precision=0.682) and medication request (recall=0.843; precision=0.685). In contrast, we observed moderate recall and high precision for medication taking (recall=0.481; precision=0.745). For provider messages, we observed excellent recall and high precision for medication question (recall=0.965; precision=0.726). Conversely, we observed low recall and moderate precision for medication question response (recall=0.342; precision=0.636). All other classes could not be predicted with this approach.

**Table 4:** Distribution of medication intent categories with examples from patient and provider messages.

Message type and medication intent category			Messages, n (%)		Example message^a^		Sublanguage features^a^
**Patient (n=282)**							
	Medication request	134 (47.5)		“Yes I am. Sent in a new prescription for the 10 mg when we changed the dosage because I *needed* to *refill* my pills.”		*need*, taking, *refill*, dose, script	
	Medication taking	79 (28.0)		“Sorry Lisinopril was *stopped HCTZ*^b^ *25 mg* added / daily Metoprolol decreased to 50 mg / *daily 25 mg started* today”		taking, *dose_freq*^c^, doseamt^d^, *din*^e^_*dose*, *started*, *stopped*, dose_treatment	
	Medication location	54 (19.1)		“You sent it to *apothecary* at 3737 *market st*?”		*apothecary_market_st*, pcam^f^_pharmacy, pah^g^_pharmacy, hup^h^_pharmacy, cvs, ravdin^i^	
	Medication question	15 (5.3)		“So at what point would / should I start 5 mg of amlodipine or another *drug?*”		*dpn*^j^*_question*, night, feeling, really_tired	
**Provider (n=252)**							
	Medication question	173 (68.7)		“Hi - we got your *refill* request - have you been taking your blood pressure medicine everyday?”		need_refilled, *refill*, *question*, taking	
	Medication question response	41 (16.3)		“I have not heard of amlodipine causing *loose stool* - if anything very rarely it can cause some *constipation*.”		typical_side_effects, *side_effect*, feel, effect_din, morning	
	Medication refill question	21 (8.3)		“Do you need refills on anything? do you need the enalipril refilled too? ok what do you *need refilled*?”		refill, refill_needed, meds^k^_need, refill_test refilled_treatment, *need_refilled*	
	Medication change	17 (6.7)		“Hi talked to Dr [**NAME**] *lets increase* your amlodipine to 10mg and see what your readings are like in a couple of weeks....”		see_tomorrow, dose_question, week, *lets_increase*, dpn_dose_program	

^a^Italics indicate encoded features identified and shared by the example sentence and sublanguage features.

^b^HCTZ: hydrochlorothiazide.

^c^freq: frequency.

^d^doseamt: dose amount.

^e^din: drug ingredient.

^f^pcam: Perelman Center for Advanced Medicine.

^g^pah: Pennsylvania Hospital.

^h^hup: Hospital of the University of Pennsylvania.

^i^ravdin: Ravdin building.

^j^dpn: drug product name.

^k^meds: medications.

**Table 5:** Performance of majority class by topic classification.

Message type and medication intent category		Recall	Precision	F1 score
**Patient**				
	Medication location	0.833	0.682	0.749
	Medication question	—^a^	—	—
	Medication request	0.843	0.685	0.756
	Medication taking	0.481	0.745	0.585
**Provider**				
	Medication change	—	—	—
	Medication question	0.965	0.726	0.829
	Medication question response	0.342	0.636	0.445
	Medication refill question	—	—	—

^a^The class could not be predicted with this approach.

## Discussion

### Principal Findings

We developed and applied an unsupervised method, LDA, to facilitate the review and derivation of patient and provider intents from a large data set of messages produced using an asynchronous, bidirectional communication platform. We learned that LDA can be leveraged to identify subgroups of messages, but with some limitations.

First, we successfully applied a data-driven approach using single-word frequencies scaled by L2-penalized regression coefficients and detected distinct word usage by role (ie, patients and providers). Patients used temporal expressions (eg, “morning” and “evening”) to initiate requests, medical terms (eg, “rx” and “pulse”) to communicate medication and BP reporting, and confirmations (eg, “okay” and “thx”) to convey information understanding. Providers commonly used salutations (eg, “mrs” and “mr”) to initiate communication with patients, directive verbs (eg, “check record,” “sign,” “send,” “confirm,” “recheck,” and “look”) to instruct the patient, and positive and negative sentiments (eg, “nice,” “great,” and “worry”) to encourage patients to continue program engagement. We did not conduct a formal sentiment analysis.

These initial insights informed the decision to develop LDA models based on roles—patient and provider—to identify potentially distinct topics among messages. In the summary overview of the 50 LDA-derived topics, the results initially seemed promising. In LDA models for both patient and provider messages, the majority of messages occurred within a few prominent major topics, and the terms comprising each topic appeared sensible. For example, among the most common topics from provider-generated messages, we observed topics with terms indicative of BP checking and reporting (ie, topic 47: “bp” and “check”), BP reporting encouragement (ie, topic 12: “good,” “work,” “keep,” and “great”), and appointment scheduling (ie, topic 42: “call,” “appt,” and “come”). Among the most common topics from patient-generated messages, we observed topics with terms suggestive of confirmation and gratitude (ie, topic 1: “thank” and “nice”), medication adherence and BP reporting (ie, topic 17: “pulse,” “mg,” “tab,” and “dose”), and BP reporting (ie, topic 7: “bp,” “record,” and “check”). However, when we set out to validate the LDA-derived topics via manual annotation, we still observed significant heterogeneity of intents within LDA-derived topics. For example, in topic 17, the patient-derived topics composed of words like “mg,” “tab,” and “dose” contained diverse messages citing medication dosage, ranging from medication adherence reports to questions about medication, as well as nonmedication-related messages (eg, BP values). The topics were also not comprehensive, as several other topics contained medication-related messages. We hypothesized that the data set might be too heterogeneous for LDA; in particular, we were concerned that the messages were too complex (ie, messages often contained more than one intent) for the effective application of LDA.

Therefore, we focused our efforts on a curated subset of the data. We manually identified messages containing a single medication-related intent and annotated them according to four different medication-related intent categories, for both patient and provider messages (4 topics each). We aimed to determine how well the medLDA model could identify these medication intents as distinct topics. We observed that, generally, for each medLDA model–derived topic, there was a dominant intent, resulting in moderate precision for some classes (ie, patient: medication location, medication request, and medication taking; provider: medication question and medication question response). However, each topic still contained heterogeneity in intent, and the distribution of intents was skewed across topics. There are several potential explanations for these observations. One is that in our manually annotated reference standard, the distribution of messages with a single medication intent were largely skewed within both the patient-generated and provider-generated medication messages. Among the provider-generated medication intents, the medication question intent was predominant (~70%) among messages. Among patient-generated medication intents, the medication request or medication-taking intents were common, and they tended to co-occur among medication LDA topics. Studies of patient portal message classification also demonstrate somewhat skewed distributions in message type [26,27]. These skewed distributions and shared common vocabulary terms may explain why the medLDA models were not able to perfectly discern each medication intent across topics. Another consideration is that in narrowing our data set to single-intent messages related to medication, there was a several factor–fold reduction in our data set. Thus, the resulting data set may have been too small for LDA to learn nuanced pattern differences.

Our findings suggest that LDA may still hold promise for automatically discerning novel topics within a large corpus of text data. However, there is likely a “just right” database for its application, one where the unit of analysis contains one or two primary intents, and different intents are well represented (ie, the database is very large). Currently, the use of LDA for analyzing text data may be limited to identifying broad differences in language use patterns, with the caveat that lexical similarity does not necessarily mean intent similarity. For example, De et al [28] applied LDA topic modeling to narrow down classes of patient messages—those relating to fatigue, prednisone, and patient visits—to identify commonly occurring themes within those message classes. Our work demonstrates that LDA is limited for providing further clinical insight. Further investigation of terms and semantic categories encoded by MedEx and Stanza provided some insights of shared and distinct concepts; however, more powerful language models might be necessary to discern intents with subtle semantic differences that are important for clinical contexts.

### Limitations and Future Work

Our pilot study has several limitations. Notably, we conducted our analysis with a single data set generated from a particular patient-provider engagement program. However, we believe that unsupervised learning approaches can be beneficial for streamlining the mining of free-text data with customization to each individual program or application. As a result, this work is important because it highlights potential approaches for incorporating unstructured learning into this process. Customizations could be achieved with a larger annotated corpus and more powerful language models. Also, our unit of analysis was each message; we could have chosen to analyze other units (eg, sentences) that could improve LDA performance. However, this would not resemble real messaging practices, which often contain more than one sentence and intent. Another limitation is that our intent categories were clinically oriented, that is, they were based on clinically actionable intents. The model may have performed differently with a different reference standard framework (eg, negative, positive, or neutral sentiment). In the future, we will develop patient-provider language models, such as Bidirectional Encoder Representations from Transformers models, that might improve our ability to capture and leverage differences between message types to improve automatic intent classification.

### Conclusions

We demonstrated how unsupervised learning can be applied to group text messages and identified medication-related messages within a bidirectional text messaging system for hypertension management. While LDA was useful in generating coarse categories, more detailed intent annotation is needed to develop reliable NLP-based intent classifiers that drive clinical actions and address subtopic heterogeneity.

## Appendix

Multimedia Appendix 1 Supplementary materials.

## Abbreviations

amlopidin: amlodipine
appt, appoint: appointment
BP: blood pressure
daili: daily
DBN: drug brand name
DDF: drug dose form
DIN: drug ingredient
DPN: drug product name
eHTN: Employee Hypertension Management Program
FREQ: frequency
gm: good morning
hr: hour
hup: Hospital of the University of Pennsylvania
HypoDetect: Hypoglycemia Detector
i2b2: Informatics for Integrating Biology and the Bedside
LDA: latent Dirichlet allocation
medLDA: medication-specific latent Dirichlet allocation
ML: machine learning
NER: named entity recognition
NLP: natural language processing
PASTE: Patient-Centered Automated SMS Tagging Engine
rx: prescription
schedul: schedule
see follow: see at follow-up
tab: tablet
thx: thanks

## Data Availability

The term list and associated probabilities within each LDA topic and medLDA topic can be found at our GitHub site [29].
