# Supplementary material for: Identifying Medication-Related Intents From a Bidirectional Text Messaging Platform for Hypertension Management Using an Unsupervised Learning Approach: Retrospective Observational Pilot Study
Source: J Med Internet Res. 2022 Jun 29;24(6):e36151. doi: 10.2196/36151 (PMC9280462; doi:10.2196/36151)

**Multimedia Appendix 1. Supplementary materials.**

Figure S1. Distribution of patient messages according to shared significant subtopics within a main topic.


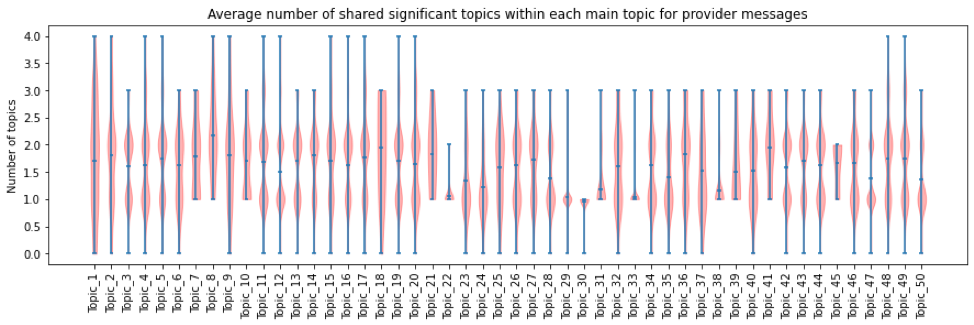


Figure S2. Distribution of provider messages according to shared significant subtopics within a main.


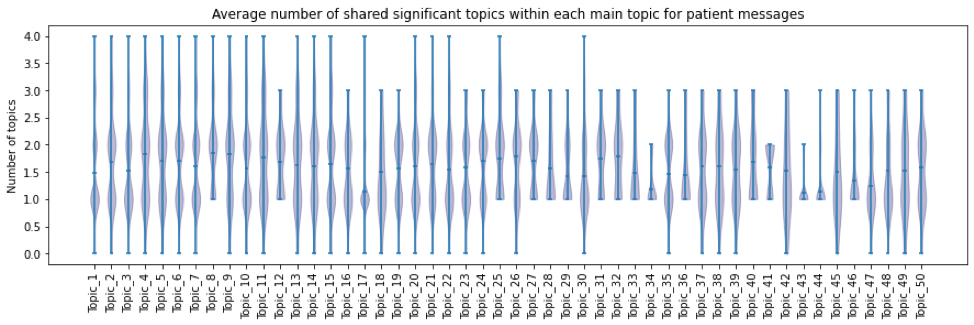

Supplement: Multimedia Appendix 1 [file jmir_v24i6e36151_app1.docx]
